# Development and evaluation of mosquito-electrocuting traps as alternatives to the human landing catch technique for sampling host-seeking malaria vectors

**DOI:** 10.1186/s12936-015-1025-4

**Published:** 2015-12-15

**Authors:** Deodatus V. Maliti, Nicodem J. Govella, Gerry F. Killeen, Nosrat Mirzai, Paul C. D. Johnson, Katharina Kreppel, Heather M. Ferguson

**Affiliations:** Institute of Biodiversity, Animal Health and Comparative Medicine, University of Glasgow, Graham Kerr Building, Glasgow, G12 8QQ UK; Environmental Health and Ecological Sciences, Ifakara Health Institute, PO Box 78373, Kiko Avenue, Mikocheni B, Dar es Salaam, Tanzania; Bioelectronics Unit, University of Glasgow, Graham Kerr Building, Glasgow, G12 8QQ UK; Department of Vector Biology, Liverpool School of Tropical Medicine, Pembroke Place, Liverpool, L3 5QA UK; School of Life Sciences, Nelson Mandela African Institute of Science and Technology Tanzania, PO Box 447, Arusha, Tanzania

**Keywords:** Mosquito electrocuting trap, Human landing catch, Mosquito behaviour, Vector sampling tools, Outdoor biting, Malaria, *Anopheles arabiensis*, *Anopheles gambiae s.l*., *Anopheles funestuss.l.*

## Abstract

**Background:**

The human landing catch (HLC) is the gold standard method for sampling host-seeking malaria vectors. However, the HLC is ethically questionable because it requires exposure of humans to potentially infectious mosquito bites.

**Methods:**

Two exposure-free methods for sampling host-seeking mosquitoes were evaluated using electrocuting surfaces as potential replacements for HLC: (1) a previously evaluated, commercially available electrocuting grid (CA-EG) designed for killing flies, and (2) a custom-made mosquito electrocuting trap (MET) designed to kill African malaria vectors. The MET and the CA-EG were evaluated relative to the HLC in a Latin Square experiment conducted in the Kilombero Valley, Tanzania. The sampling consistency of the traps across the night and at varying mosquito densities was investigated. Estimates of the proportion of mosquitoes caught indoors (P_i_), proportion of human exposure occurring indoors (π_i_), and proportion of mosquitoes caught when most people are likely to be indoors (P_fl_) were compared for all traps.

**Results:**

Whereas the CA-EG performed poorly (<10 % of catch of HLC), sampling efficiency of the MET for sampling *Anopheles funestuss.l.* was indistinguishable from HLC indoors and outdoors. For *Anopheles gambiae s.l.*, sampling sensitivity of MET was 20.9 % (95 % CI 10.3–42.2) indoors and 58.5 % (95 % CI 32.2–106.2) outdoors relative to HLC. There was no evidence of density-dependent sampling by the MET or CA-EG. Similar estimates of P_i_ were obtained for *An. gambiaes.l*. and *An. funestus s.l.* from all trapping methods. The proportion of mosquitoes caught when people are usually indoors (P_fl_) was underestimated by the CA-EG and MET for *An. gambiae s.l*., but similar to the HLC for *An. funestus*. Estimates of the proportion of human exposure occurring indoors (π_i_) obtained from the CA-EG and MET were similar to the HLC for *An. gambiae s.l*., but overestimated for *An. funestus.*

**Conclusions:**

The MET showed promise as an outdoor sampling tool for malaria vectors where it achieved >50 % sampling sensitivity relative to the HLC. The CA-EG had poor sampling sensitivity outdoors and inside. With further modification, the MET could provide an efficient and safer alternative to the HLC for the surveillance of mosquito vectors outdoors.

**Electronic supplementary material:**

The online version of this article (doi:10.1186/s12936-015-1025-4) contains supplementary material, which is available to authorized users.

## Background

Efforts to control malaria rely heavily on the application of long-lasting insecticidal nets (LLINs) which are the major strategy to protect humans against bites from mosquito vectors in African homes [1]. Rapid increases in the coverage of LLINs over the past decade have been associated with substantial declines in major African vector species [2]. A parallel decline in malaria infection rates in people has been reported in several places, as has a decrease in malaria mortality in infants and adults [3]. However, the widespread use of these vector control measures may be triggering changes in the ecology and genetics of mosquito populations that could threaten their continued effectiveness [4–7].

Insecticide resistance is increasingly reported in areas where LLINs are widely used [8–11]. There are also concerns that LLINs may be selecting for behavioural changes within malaria vectors that allow them to shift their biting to times and places where people are not protected, which can be defined as ‘behavioural avoidance’ [7, 12–16]. These changes in feeding behaviours could arise either due to shifts in malaria vector species composition from dominance by highly endophilic and anthropophilic species (e.g., *An. gambiaes.s.*) towards those with more exophilic and zoophilic tendencies such as *Anopheles arabiensis* [17]. Additionally, it has been hypothesized that selection from LLINs could generate within-species behavioural adaptations [13, 15, 18, 19]. The ability to monitor if and how rapidly mosquito behaviour is changing in response to control measures is crucial for assessment of the continued effectiveness of LLINs and indoor residual spraying (IRS) strategies [20–23].

One of the most important and widely used techniques to study the host-seeking behaviour of mosquitoes is the human landing catch (HLC) technique. This technique is regarded as the gold standard tool for sampling host-seeking malaria vectors [24, 25]. The HLC is widely used for a range of purposes, including estimation of entomological exposure rates [26–29], evaluation of vector control measures [30, 31] and for studying mosquito vector behaviour and ecology [16, 26–28, 32, 33]. Although the HLC provides a realistic estimate of the number of mosquito bites that humans are exposed to, this technique has numerous drawbacks. The most notable is ethical concerns raised by requiring the participating human subjects to expose their legs to attract mosquitoes. The aim is for participants to capture mosquitoes landing on them before they bite, but this is not always possible and could generate some risk of exposure to infection [26, 34, 35]. To minimize exposure risk it is recommended that HLC participants use malaria prophylaxis [36]. Whilst it has been shown this precautionary measure can reduce infection risk in HLC participants to below that experienced in the community in some settings [36], it is unlikely to be effective in areas of high drug resistance, and/or where mosquitoes carry other pathogens (e.g., dengue, filariasis) that pose infection risks [37]. These problems highlight the need for a more efficient, representative and ethical alternative sampling method for investigation of mosquito biting densities and behaviour.

Previous attempts have been made to develop exposure-free sampling tools for collecting indoor or outdoor biting mosquitoes. These techniques include but are not limited to the bed net trap [38], tent traps [39–41], the CDC light traps [42], and the mosquito magnet (MM) trap [43–45]. While these methods have shown promise in some settings, most have limitations that restrict their large-scale application, and/or bias collection towards mosquito species with particular phenotypes that may misrepresent the community of mosquitoes attracted to people [46]. Recently, there has been renewed interest in exploring the use of electrocuting surfaces as a means of sampling malaria vectors [47–49]. This approach was originally developed for trapping tsetse flies outdoors [50], but later adapted to sample mosquitoes drawn towards a host odour source [51, 52]. This trap works by placing a live host in a sealed tent and piping their odour out to an electrocuting net (E-Net) approximately 10 m away that kills mosquitoes on contact. Such E-Nets have already shown promise when used to investigate host species’ preferences and odour responses of the African vector species [51, 52]. As a potential improvement, the use of commercially available ‘bug-zapping’ devices, which can sample insects in the immediate proximity of a host has been explored with some promise, indicating they can achieve a relative sampling efficiency of up to 50 % of the HLC in one study [49]. However, given these devices were developed for large flies, their suitability for trapping African malaria vectors is unclear. Further work is required to develop an electrocuting trap that is optimized for malaria vectors, can meet the performance of the HLC, is suitable for use inside and outdoors, can be used safely in close proximity of humans, and is durable under field conditions. Here, a mosquito electrocuting trap (MET) was designed, developed and field-tested. This trap was custom designed to sample host-seeking African malaria vectors, with the aim of meeting all performance targets defined above.

## Methods

### Study site

Field experiments were conducted at Lupiro village (−8.38 S, 36.67 E) located in the Kilombero Valley of southern Tanzania. This village is situated in a malaria-endemic region where the most recent estimate of entomological inoculation rate (EIR) was 33.9 infectious bites per person per year [53]. Historically *An. gambiae s.s.* was the most abundant member of the *An. gambiaes.l.* species complex in the Kilombero Valley [54]. However in conjunction with the increasing coverage of insecticide-treated bed nets (ITNs) in this area over the last decade [55, 56], *An. gambiae s.s.* has almost virtually disappeared and its sibling species *An. arabiensis* now constitutes >98 % of the species complex in most areas [57–59]*. Anopheles funestus* is the only other important vector species in the area [60].

### Trapping methods

**Fig. 1:**
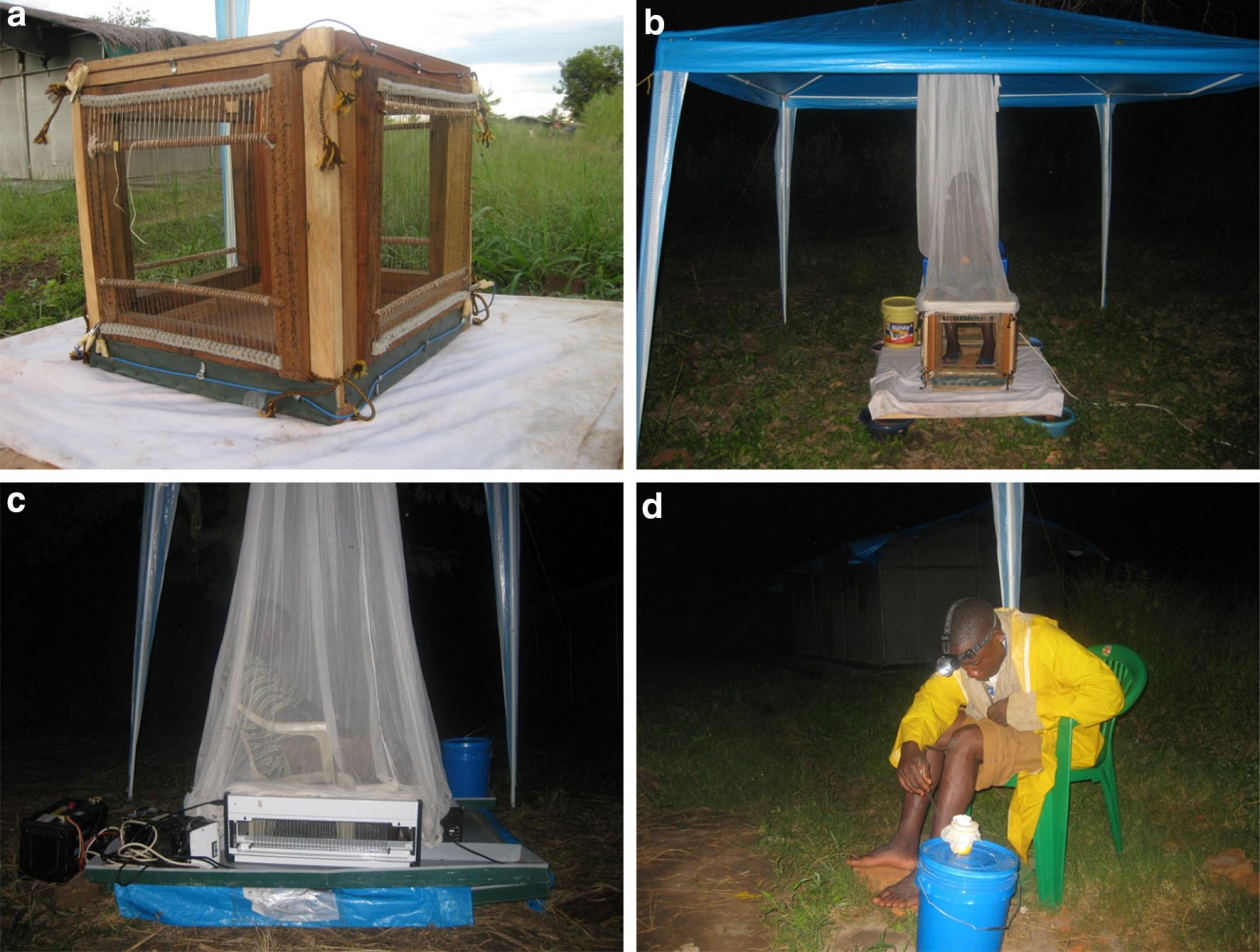
Deployment of traps. **a** The MET, **b** the MET with a person sitting with his legs in the trap, **c** a person sitting within the CA-EG trap and **d** a person performing a human landing catch

Three different trapping methods were used in this experiment: the HLC, a MET developed in collaboration between the Ifakara Health Institute (IHI) and the University of Glasgow, and a commercially available ‘bug zapper’ device (PlusZap™ model ZE107 PZ40W [61]; defined as the CA-EG in this study) which is sold for domestic electrocution of insects [49]. The MET consists of four 30 × 30 cm panels connected together to make a square trapping box. On each panel, a mesh grid was made by placing ~1 mm thick (stainless steel) wires parallel to one another, at a spacing of 5 mm. Adjacent wires were supplied with opposite electric charge (positive next to negative) from a common positive or negative electric terminal. Wires were fixed into a wooden frame, with the four wooden frames being attached together to make the trap (Fig. 1a). The space between adjacent wires was set at 5 mm because this was deemed to be sufficiently small to prevent a mosquito from flying through, but ensure a mosquito made contact with both a negative and positive grid resulting in electrocution. *Anopheles gambiae s.l.* wing size, which was used to decide the spacing between the grids, was estimated from [62]. To collect mosquitoes using a MET, a person sits with their lower legs positioned inside the trapping box (Fig. 1b). This set-up imitates the HLC with the intention of making the MET as closely efficient to the HLC as possible. The CA-EG trap, with one of its four panels having surface dimensions of 68 × 24 cm (Fig. 1c), electrocutes flying insects on contact with the electrified surface, which is made up of grid wires placed about 8 mm apart. The adjacent wires of the electrocuting grid are connected to positive and negative terminals at ~800 V alternating current (AC). Four panels of the CA-EG were joined to form a square structure (Fig. 1c) into which a human sitting on a chair placed their legs. Mosquitoes which were attracted to bite the person on the legs were electrocuted on contact with the electrified grids. In both MET and CA-EG, the remaining part of the catchers were not surrounded by the electric grid but were protected by netting.

This experiment was conducted using a series of experimental huts designed to imitate the typical design of local houses in the study area [63]. Experimental huts dimensions were 6.5 m long × 3.5 m wide × 2 m high, with a 20-cm wide gap between the top of walls and roof to simulate the open eaves found in most local houses. Trapping stations were set up inside each hut and at an associated outdoor point approximately 10 m away. For outdoor stations, tents were used to provide roofing that protected the traps and collectors from rain (Fig. 1). A 3 × 3 Latin square design was used in which each of the three trapping methods was randomly assigned to one of three experimental huts on each night. On each night, collections were conducted at paired indoor and outdoor trapping stations. Over consecutive nights, the three trapping methods were trialled at each hut to complete a full rotation in 3 days. Seven rounds of trapping were conducted over 21 trapping nights between March and May 2012. The first two rounds were conducted in a group of three experimental huts defined as A, B and C (at site 1), and the remaining five rounds were conducted in a different group of experimental huts (defined D, E and F) which were situated approximately 200 m from the first.

Trapping was conducted from 19.00 to 07.00 hours. During each hour, volunteers spent 45 min passively sitting in a trap (MET or CA-EG) or actively collecting mosquitoes (HLC, Fig. 1d), with the remaining 15 min used as a break. Collectors moved to a different trap or position every hour throughout the night to minimize bias due to variation in their relative attractiveness to mosquitoes. At the end of each hour, MET and CA-EG traps were checked and trapped mosquitoes were removed by mouth aspirators or forceps and placed in labelled cups. On the following morning, mosquitoes from all the three trapping methods were sorted using morphological keys to identify their genera and gender. Female mosquitoes visually identified as belonging to a malaria vector group (*An. gambiaes.l*. or *An. funestus s.l.*) were individually stored in Eppendorf tubes with silica gel. *Anopheles gambiaes.l.* were later analysed using the polymerase chain reaction (PCR) technique to identify their species identity [64].

### Statistical analysis

All statistical analyses were carried out using the R statistical software version 2.15. Generalized linear mixed models (GLMMs) [65] were used to assess variation in mosquito vector abundance between trap types. Mosquito abundance data were highly overdispersed and thus modelled as following a negative binomial distribution using the generalized linear mixed model automatic differentiation model builder (glmmADMB) package [66]. Here, trap type was fitted as the primary main effect of interest, and experimental night and hut as random effects. The relative sampling efficiency of the novel trap types relative to the HLC was estimated by computing the ratio of the predicted nightly abundance of vectors from these statistical models.

To test whether there was any systematic increase or decrease in the sampling efficiency of the CA-EG or MET relative to the HLC over the course of a night (e.g., perhaps due to battery decline), a model was constructed in which the fraction of the hourly catch occurring either in CA-EG or MET (e.g., ‘novel trap’/(‘novel trap + HLC’) was defined as the response variable, and trapping hour (defined as being ‘1’ on the first hour, and increasing through the night to 12 as the last hour) fitted as a continuous fixed effect, with experimental night added as a random effect. Here, the proportion of mosquitoes caught by a novel method (CA-EG or MET) out of the total caught from this method and the HLC, was modelled using a GLMM using a logit link function.

Additional analysis was conducted to test whether the relative performance of the novel trap types was density dependent. Density dependence was investigated using the Bland–Altman method which assesses the reliability of two measures via regression analysis of the relationship between their difference and their mean [67], where non-linearity in this relationship indicates density dependence (see Additional file 1). Values of *R*_adj_^2^ obtained from these analyses can be interpreted as an estimate of the proportion of deviation from perfect linear correlation due to density dependence rather than random error (with a high value indicating support for density dependence). The precision of the *R*_adj_^2^ estimate was gauged by estimating its 95 % confidence interval as the 2.5th and 97.5th centiles from 10,000 bootstrap replicates.

Finally, analyses were conducted to assess if the three focal trapping methods varied in their prediction of key mosquito vector behaviours and their related human exposure outcomes [68]. The predictors of malaria vector type of behaviour that were analysed here are the proportion of mosquitoes that were caught feeding indoors (P_i_), the proportion of mosquitoes that were caught feeding when most people were indoors (P_fl_), and the proportion of human exposure that occurs indoors (π_i_) [49, 68–70]. The proportion of mosquitoes that were caught indoors (P_i_) was calculated by dividing the total number of mosquitoes caught indoors by the total number caught outdoors and indoors over 12 h of the night: I_19→07 h_/(I_19→07 h_ + O_19→07 h_) [70], where I and O, respectively, represent mosquitoes collected indoors and outdoors, and subscripts indicate the start and the end time of the sampling period. The calculation of P_fl_ and π_i_ requires definition of the period of the night when most people (>50 %) are expected to be indoors and sleeping. This time period was previously estimated for the community living in Lupiro village as 21.00–05.00 [71]. Therefore, the proportion of mosquitoes caught when most people were likely to be indoors (P_fl_) was calculated as follows: (I_21→05 h_ + O_21→05 h_)/(I_19→06 h_ + O_19→06 h_) [70]. The proportion of human exposure that occurs indoors (πi) was calculated by dividing the number of mosquitoes caught indoors during the period that most people are inside (21.00–05.00) by itself plus the number of mosquito caught outdoors outside of the sleeping hours (I_21→05 h_)/(I_21→05 h_ + O_19,20,06 h_) [70]. Binary estimates of P_i_, P_fl_ and π_i_ were estimated using GLMMs with a binomial distribution and a logit link function [65]. In these models, trap type was fitted as a fixed effect, and experimental night as a random effect.

### Ethical procedures

Ethical approval was obtained from the Institutional Review Board of the Ifakara Health Institute (Reference number IHI/IRB/A.50) and the Medical Research Coordination Committee of the National Institute for Medical Research, Tanzania (Reference number NIMR/HQ/R.8a/Vol. IX/801.) All volunteers recruited in this work were given informed consent forms with details of the procedures, explanation of their right to withdraw at any time, potential risks, and mitigation plan. All participants read and signed the forms before taking part in the work. All participants were provided with malaria prophylaxis, Malarone (250 mg atovaquone and 100 mg proguanil hydrochloride, GlaxoSmithKline) before and during the experiments to prevent malaria infection.

## Results

Over all 21 nights of experiments, 18,497 mosquitoes were collected representing five genera comprising *Anopheles*, *Culex*, *Mansonia*, *Aedes*, and *Coquillettidia* (Table 1). Seven *Anopheles* species were sampled of which *An. gambiae s.l.* was the most abundant. Four-hundred of the 5559 *An. gambiae s.l.* sampled were individually tested using PCR, and all were found to be *An. arabiensis*. This observation matches other recent reports indicating *An. arabiensis* constitutes more than 98 % of the *An. gambiae s.l.* species complex in Lupiro [57–59]. As malaria vectors were the prime focus of interest in this study, all further analyses are restricted to female *An. gambiae s.l*. and *An. funestuss.l.*

**Table 1:** A summary of the total number of mosquito genera and species caught by different sampling methods in this study

Taxon	Total per trapping method			Female	Male	Total	% Composition
	HLC	MET	CA-EG				
*An. gambiae s.l*.	3443	1786	330	5559	5	5564	30.08
*An. funestus s.l.*	772	650	121	1543	13	1556	8.41
*An. coustani*	664	49	26	739	0	739	4.00
*An. pharaoensis*	10	4	3	17	0	17	0.09
*An. squamosus*	46	19	14	79	0	79	0.43
*An. wellcomei*	5	1	1	7	0	7	0.04
*An. ziemani*	353	32	6	391	0	391	2.11
*Culex*	1815	829	383	3027	129	3156	17.06
*Mansonia*	3829	1429	1603	6861	64	6925	37.44
*Aedes*	10	1	0	11	0	11	0.06
*Coquillettidia*	30	12	5	47	2	49	0.26
Grand total	10,977	4812	2492	18,284	213	18,497	100.00

*HLC* human landing catch, *MET* mosquito electrocuting trap, *CA-EG* commercially available electric grid trap

### Sampling sensitivity

**Fig. 2:**
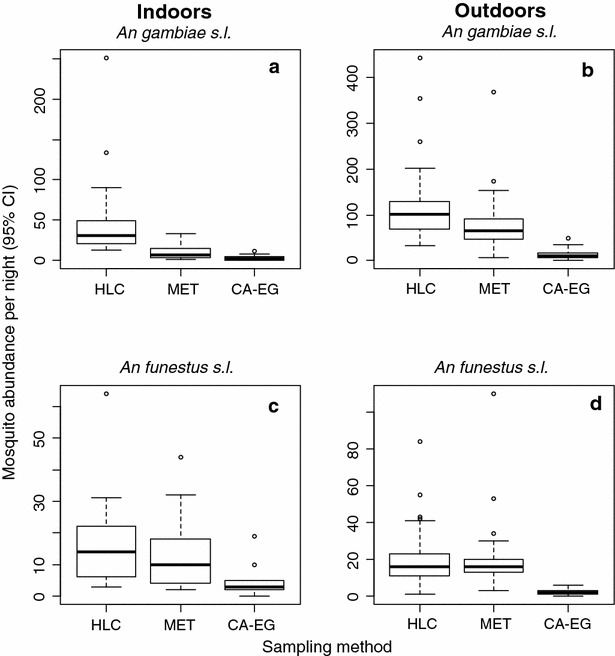
A *box plot* of raw mosquito abundance per sampling night as caught by each of the three sampling methods

**Fig. 3:**
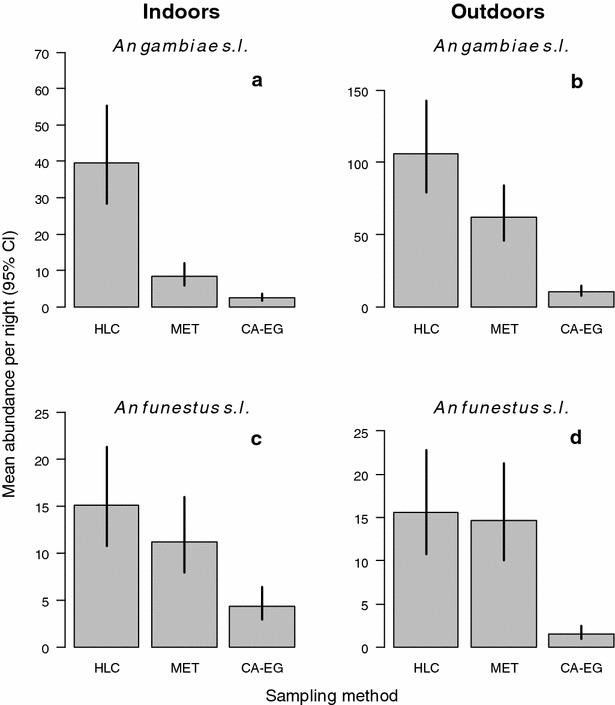
The mean abundance of malaria vector species caught per sampling night for each of the three traps evaluated. *HLC* human landing catch, *MET* electric grid trap, *CA-EG* commercially available electric grid trap. *Bars* represent 95 % confidence interval

Approximately 3.5 times more *An. gambiaes.l.* (N = 5559) were collected than *An. funestuss.l.* (N = 1543, Table 1), with more of both species being sampled outdoors than indoors (Fig. 2). The sampling sensitivity of traps varied between indoor and outdoor environments for both *An. gambiae s.l.* (trap × location interaction: χ_2_^2^ = 253.4, *p* < 0.001) and *An. funestus s.l.* (trap × location: χ_2_^2^ = 9.0, *p* = 0.003). Regardless of location (indoor vs out), the HLC consistently sampled significantly more *An. gambiae s.l.* than either the MET (outdoors: z = 4.10, *p* < 0.001; indoors: z = 7.89, *p* < 0.001) or the CA-EG (outdoors: z = 16.00, *p* < 0.001, indoors: z = 11.99, *p* < 0.001, Fig. 3a, b). There was significant variation between the electrocuting traps, with the MET catching significantly more *An. gambiae s.l.* than the CA-EG both indoors (z = 4.89, *p* < 0.001, Fig. 3a) and outdoors (z = 12.4, *p* < 0.001, Fig. 3b). Based on these results, the sampling efficiency of the MET relative to HLC for *An. gambiae s.l.* was estimated to be 59 % outdoors, and 21 % indoors (Table 2). The sampling efficiency of the CA-EG achieved <10 % of the HLC indoors and out (Table 2). The number of *An. funestuss.l.* caught per night by the HLC and MET was not significantly different either when used indoors (z = 1.71, *p* = 0.09, Fig. 3c) or outdoors (z = 0.58, *p* = 0.56, Fig. 3d). In contrast, the CA-EG caught significantly fewer *An. funestuss.l.* than either the HLC or MET (*p* < 0.001 for indoors and outdoors, Fig. 3c, d). Overall, the CA-EG had a sampling efficiency of <30 % for *An. funestuss.l.* relative to both HLC and MET (Table 2).

**Table 2:** Predicted sampling efficiency of the novel traps (per night) relative to the HLC gold standard

Species	Location	Trap	Relative sampling efficiency (95 % CI)	*p* value
*An. gambiae s.l.*	Indoors	MET	20.9 (10.3–42.2)	<0.001
		CA-EG	6.1 (2.8–13.1)	<0.001
	Outdoors	MET	58.5 (32.2–106.2)*	0.55
		CA-EG	9.9 (5.3–18.4)	0.023
*An. funestus s.l.*	Indoors	MET	74.2 (37.0–148.9)*	0.12
		CA-EG	28.7 (13.9–59.6)	<0.001
	Outdoors	MET	93.5 (43.9–199.3)*	0.86
		CA-EG	9.6 (4.0–22.7)	0.90

Asterisks are placed in cases where the upper limit of the 95 % confidence interval includes 100 %, indicating no significant difference between the performance of a novel trap compared to the HLC

### Sampling consistency across the night

**Fig. 4:**
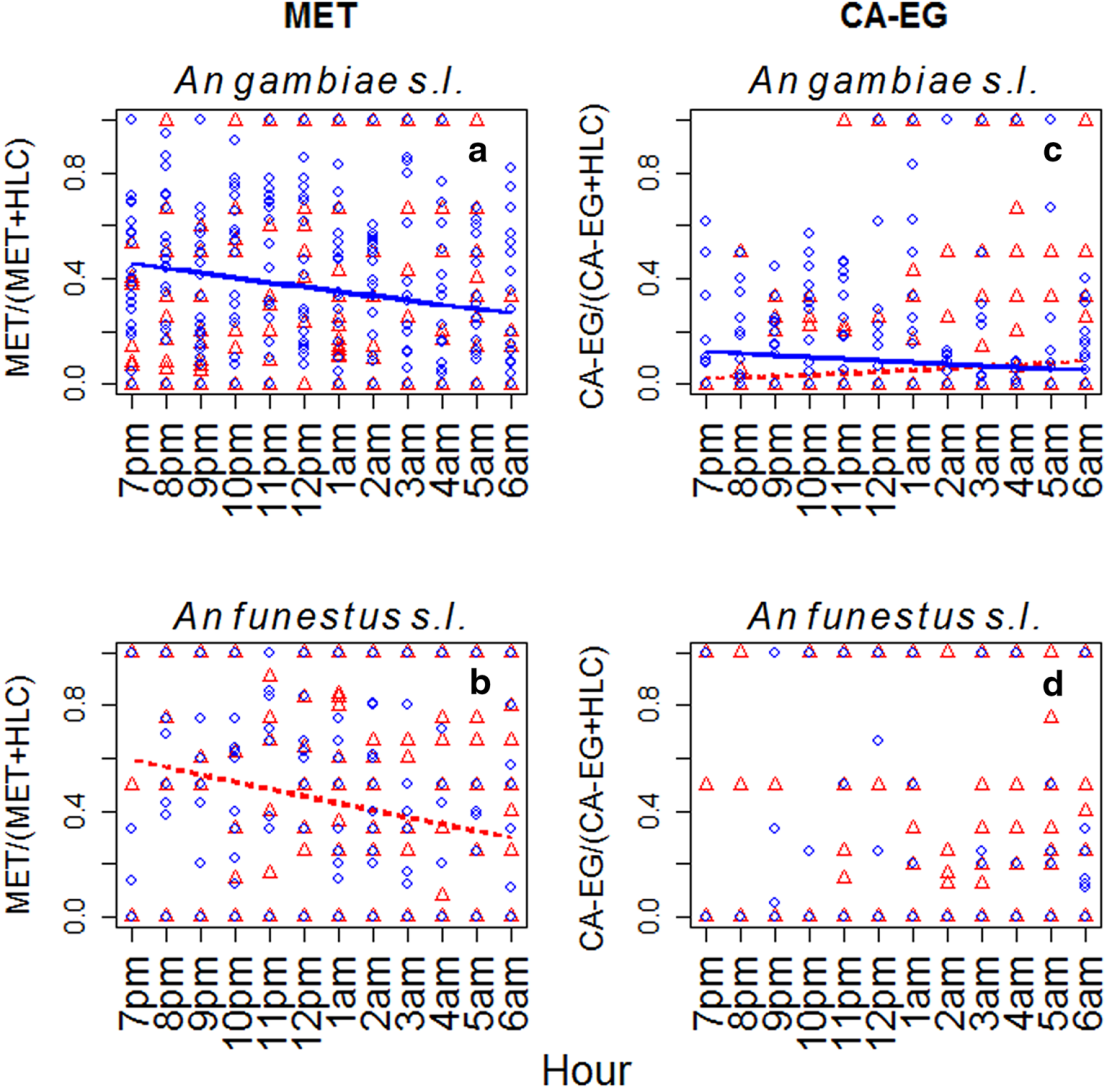
The sampling efficiency of the two novel trap types (CA-EG and MET) relative to the HLC gold standard across the hours of a sampling night. Points indicate the proportion of the total catch (new trap + HLC) that was captured by the new trap over each hour of a sampling night (19.00–07.00). *Triangle symbols* are for collections made indoors, and round dots for outdoors. *Dotted-red* and *solid-blue lines* represent predicted relationship between the relative sampling efficiency across the hours of a sampling night, indoors and outdoors, respectively, (*lines* only shown when there was a statistically significant change through time)

The sampling efficiency of the MET relative to the HLC remained constant across the hours of the night when used for *An. gambiae s.l.* indoors (χ_1_^2^ = 0.001, *p* = 0.98), however there was evidence of a moderate decline through time when used outdoors (χ_1_^2^ = 52.11, *p* < 0.001, Fig. 4a). This trend was reversed for *An. funestus s.l.* where the sampling efficiency of the MET relative to the HLC was observed to decline somewhat over the sampling night indoors (χ_1_^2^ = 12.42, *p* < 0.001, Fig. 3b), but remained stable outdoors (χ_1_^2^ = 0.76, *p* = 0.38). The sampling efficiency of the CA-EG relative to the HLC showed some increase across the night when used to sample *An. gambiae s.l.* indoors (χ_1_^2^ = 10.36, *p* = 0.001, Fig. 4c), but declined outdoors (χ_1_^2^ = 17.42, *p* < 0.001, Fig. 4c). The sampling efficiency of the CA-EG relative to the HLC for *An. funestuss.l.* was constant across the night both indoors (χ_1_^2^ = 0.39, *p* = 0.54, Fig. 4d) and outdoors (χ_1_^2^ = 2.31, *p* = 0.13, Fig. 4d).

### Sampling consistency across varying mosquito densities

**Fig. 5:**
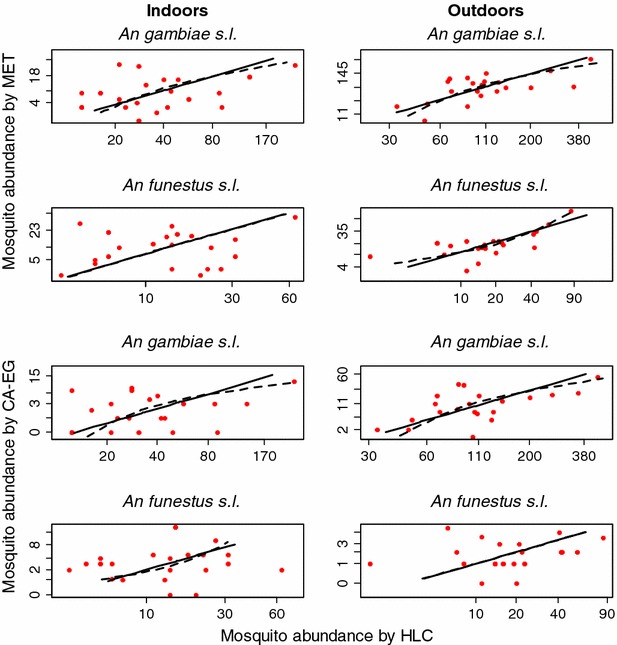
Assessment of non-linearity as a measure of density dependence of the electrocuting traps relative to the HLC. *Solid lines* represent a model of linear relationship between the numbers of mosquitoes collected by a novel trap relative to HLC, while *dotted lines* were obtained from non-linearity, which was modelled as a natural cubic spline with two degrees of freedom

In general, there was a positive association between the number of malaria vectors caught per night in the MET and the HLC, although the Pearson linear correlation coefficients were not statistically significant in all cases (Additional file 2: Table S2). A similar pattern of positive, but not always statistically significant, correlations between CA-EG and HLC catches was observed (Additional file 2: Table S2). Nightly catches were log(x + 1) transformed and plotted for further investigation of potential density dependence as evidenced by deviation from linearity. In all cases, there was much stronger support for a linear relationship between the log-transformed values of nightly catches than a curvilinear association (Fig. 5). All of the estimates of the strength of density dependency (adjusted *R*^2^) were close to zero, but often with a wide confidence interval ranging from below zero to above 40 % in some cases (Table 3), suggesting that power to detect low-to-moderate levels of density dependence was limited. However, based on the range of mosquito densities encountered in this trial, there is no evidence to indicate the relative performance of the CA-EG or MET is density dependent when used indoors or outside.

**Table 3:** Quantification of density dependence using the Bland–Altman method

Taxon	Location	Method	Adjusted R^2^ (95 % CI)	*p* value
*An. gambiae* s.l.	Indoors	MET	−9 (−11, 35)	0.55
		CA-EG	−6 (−11, 27)	0.36
	Outdoors	MET	−1 (−9, 44)	0.20
		CA-EG	−3 (−9, 36)	0.25
*An. funestus* s.l.	Indoors	MET	−11 (−11, 16)	0.95
		CA-EG	−9 (−11, 34)	0.64
	Outdoors	MET	−1 (−9, 62)	0.20
		CA-EG	−11 (−11, 40)	0.95

Adjusted R^2^ values show estimates of the proportion of deviation from perfect linear correlation that is likely to be due to density dependence rather than random error. As adjusted R^2^ values are penalized for model complexity, negative estimates are possible, but should be interpreted as zero

### Metrics of mosquito behaviour and human biting exposure distribution

**Fig. 6:**
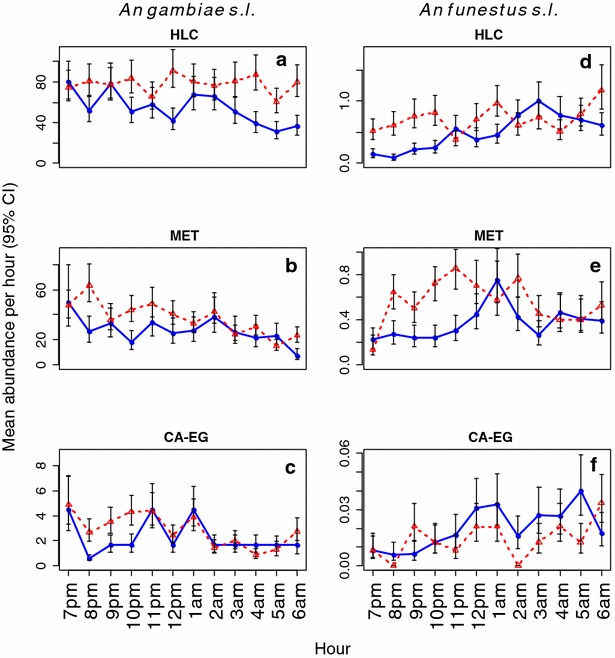
Predicted mean abundance of *Anopheles gambiae s.l.* and *Anopheles funestus* *s.l.* for each hour of the night, in indoor and outdoor environments. *Dotted-red* and *solid-blue lines* show predicted mean abundance of mosquito across the night for outdoor and indoor locations, respectively. *Error bars* represent 95 % confidence intervals

Mosquito hourly biting activity was quite variable between nights, and revealed no obvious peaks in biting times for either *An. gambiaes.l.* or *An. funestuss.l.* (Fig. 6). All traps indicated that *An. gambiaes.l.* was significantly exophilic (>60 % of bites taking outdoors), while *An. funestuss.l*. was estimated to bite indoors and outdoors at similar rates (~50:50 split between indoor and outdoor biting, Table 4). Estimates of the proportion of *An. gambiaes.l.* that feed indoors (P_i_) obtained from the HLC and MET were similar (z = −1.15, *p* = 0.25, Table 4), as were those obtained from the HLC and CA-EG (z = −1.77, *p* = 0.08, Table 4). Estimates of the proportion of indoor biting in *An. funestus s.l.* were also similar between the HLC and MET (z = 1.65, *p* = 0.10, Table 4), and the HLC and CA-EG (z = 1.95, *p* = 0.051, Table 4).

**Table 4:** Indicators of malaria vector biting behaviour and human exposure metrics (P_i_, P_fl_ and π_i_) as estimated by each of the three traps for *Anopheles gambiae s.l.* and *Anopheles funestus s.l.*

Taxon	Method	Proportion caught indoors (P_i_)		Proportion caught when most people are indoors (P_fl_)		Proportion of human exposure occurring indoors (π_i_)	
		Estimate (95 % CI)	*p*	Estimate (95 % CI)	*p*	Estimate (95 % CI)	*p*
*An. gambiae* *s.l*.	HLC^a^	0.37 (0.34–0.40)	N/A	0.93 (0.89–0.96)	N/A	0.43 (0.37–0.50)	N/A
	MET	0.35 (0.32–0.38)	0.25	0.68 (0.56–0.79)	<0.001	0.36 (0.31–0.42)	0.042
	CA-EG	0.34 (0.31–0.37)	0.08	0.40 (0.28–0.54)	<0.001	0.47 (0.40–0.53)	0.44
*An. funestus* *s.l.*	HLC^a^	0.51 (0.47–0.56)	N/A	0.76 (0.68–0.82)	N/A	0.55 (0.48–0.63)	N/A
	MET	0.51 (0.46–0.55)	0.10	0.70 (0.62–0.77)	0.18	0.74 (0.68–0.80)	<0.001
	CA-EG	0.55 (0.47–0.64)	0.51	0.63 (0.53–0.71)	0.009	0.81 (0.74–0.86)	<0.001

Estimates of the proportion of mosquitoes caught when most people are indoors (P_fl_) and the proportion of human exposure occurring indoors (π_i_) were calculated based on mosquito numbers collected during times when most people are indoors (21.00–05.00)

The *p* values listed are tests of the comparison of the estimates obtained from the electrocuting traps and those from the HLC (as the reference trap)

^a^Reference trap

The electrocuting traps were less consistent with the HLC when used to estimate other human exposure indicators. The HLC predicted that approximately 98 % of *An. gambiae s.l.* attempted to feed during hours when most people would be indoors (P_fl_, Table 4), which was underestimated at 68 % (z = 9.27, *p* < 0.001, Table 4) and 40 % (z = −12.91, *p* < 0.001, Table 4) by the MET and CA-EG, respectively. Predictions were less variable for *An. funestuss.l.*, where P_fl_ was estimated to be ~70–75 % by the MET and HLC, respectively (z = −1.34, *p* = 0.18, Table 4), but underestimated as 65 % by the CA-EG (z = −2.62, *p* = 0.009, Table 4). It is noted that values of P_fl_ were underestimated in all scenarios where the novel trap type (CA-EG or MET) had a lower sampling sensitivity inside than outside. The MET somewhat underestimated the proportion of human exposure occurring indoors (π_i_ = 36 %) in comparison to the HLC for *An. gambiaes.l.* (43 %), a difference of borderline statistical significance (z = −2.04, *p* = 0.04). Estimates of π_i_ for *An. gambiaes.l.* as obtained from the CA-EG and HLC were indistinguishable (43–46 %, z = 0.77, *p* = 0.44, Table 4). Both the MET (z = 4.21, *p* < 0.001) and CA-EG (z = 5.23, *p* < 0.001) overestimated π_i_ for *An. funestuss.l.* (73–80 % Table 4) compared to the HLC (55 %, Table 4).

## Discussion

In this study, the potential of two electrocuting traps, the MET and CA-EG, to provide exposure-free alternatives to the HLC technique for sampling African malaria vectors was evaluated. The HLC generally collected more *An. gambiae s.l*. than the MET, but capture rates of *An. funestus s.l.* were similar between these methods. The relative sampling efficiency of the MET was reasonably high (~59 %) when used for *An. gambiae s.l.* outdoors, but fell to ~20 % relative to the HLC when applied indoors. In contrast, the CA-EG performed poorly relative to the HLC in both indoor and outdoor settings, for *An. gambiae s.l*. and *An. funestus.* No evidence of density-dependent sampling was observed in either electrocuting trap. Both the MET and CA-EG tended to have higher performance relative to the HLC outdoors compared to indoors, which contributed to these traps producing somewhat biased estimates of human exposure indices. While estimation of the proportion of mosquitoes caught indoors (P_i_) by the electrocuting traps were similar to those estimated by HLC, there was tendency of the MET and CA-EG to underestimate (P_fl_) when sampling *An. gambiaes.l.*, and overestimate the proportion of human exposure that occurs indoors (π_i_) when sampling *An. funestuss.l.* On balance, the sampling sensitivity of the CA-EG was judged too low to merit further consideration as an alternative to the HLC. However, the MET showed strong promise as an alternative method for exposure-free surveillance of African malaria outdoors outside of houses.

The sampling efficiency of the MET was consistently higher for *An. funestus s.l.* than for *An. gambiae s.l.* Possible explanations for this include differential sensitivity of these species to electrocution. Several biological factors are known to influence the electrical conductivity of insects, including their cuticular hydrocarbon composition [72], body size and water content. Differential electrical conductivity between mosquito species could be expected to be less consequential at higher voltages say 50,000 V as used in [51] because this voltage would be used with lower currents. The voltage and current combination used in the MET were optimized in laboratory studies to produce a high instant kill rate (>80 %) using *An. gambiaes.s.* as a model, but may be more efficient at killing *An. funestuss.l*. A previous study using the CA-EG found that sampling efficiency varied between *An. gambiae s.s*. and *An. arabiensis* [49], thus vector-specific sampling may be a common feature of electrocuting traps as has been documented with other methods, such as CDC light traps [73].

Both electrocuting traps had higher sampling efficiency when used outside than indoors. The reasons underlying this are unknown but could be due to microclimatic variation [74] which could modify the functioning of electrocuting traps in outdoor and indoor settings, and/or differences in how vectors host seek in outdoor versus indoor location. For example, factors such as the direction and concentration of host odours and wind movement vary between indoor and outdoor settings [74], and could lead to differential attractiveness of the traps when used in different places. Humans conducting HLC usually bend to collect mosquitoes landing on their legs as shown in Fig. 1d, blowing carbon dioxide to the legs therefore attracting more mosquitoes when doing HLC compared to MET and CA-EG in which carbon dioxide is blown away (Fig. 1b, c). This phenomenon is expected more pronounced indoors than outdoors where wind may blow away the carbon dioxide and may therefore explain a poorer performance of MET and CA-EG indoors relative to the HLC. HLC may not therefore be a perfect indicator of mosquito-biting activities as the stated phenomenon above may bias its function. Further investigation of the performance of electrocuting traps in a broader range of ecological settings is required, including experiments that involve mechanisms to control the breath of the catchers sitting on the HLC as well as on the MET, perhaps by using a breathing tube which directs the carbon dioxide away from traps or towards the traps to increase sensitivity of both methodologies.

There were differences in the relative sampling sensitivity of CA-EG as estimated in this study compared to that reported by Majambere et al. [49]. Whereas [49] estimated the sampling efficiency of the CA-EG to be ~50 % relative to the HLC in indoor and outdoor locations, it was only 6–29 % in this study. One explanation could be variation in how human participants were positioned. In [49] the human bait lay down and were covered by bed nets which were surrounded by six grid units, in this current study the humans were positioned in a sitting position using four grid units, specifically to replicate the human subject’s position in the HLC technique and thus avoid bias due to differential positioning of the hosts. Enclosing the whole human in the trap as was done by [49] may have contributed to their higher reported performance of the CA-EG in their study compared to this study. Another difference was that the study by [49] was conducted in Dar es Salaam where *An. gambiaes.s*. is the dominant species, compared to *An. arabiensis* in the Kilombero Valley where this study was set. During preliminary laboratory optimization tests conducted during the development of the MET, *An. gambiaes.s*. was shown to be somewhat more sensitive to electrocution than *An. arabiensis.* Thus, the lower performance of the CA-EG in the current study may also be due to differences in malaria vector species composition between sites.

One of the ways to make MET smaller and therefore easy to carry around would be to replace the human bait with an artificial odour delivery system. This step would additionally remove human safety concerns and significantly decrease labour. However, to be able to obtain an alternative trapping tool with sampling efficiency close to the gold standard HLC, this study tried to imitate as much as possible some of the features which make HLC superior to other host-seeking traps. Theoretically, a good host-seeking trap should represent as much as possible human exposure rates to host-seeking mosquitoes that happen in real environment. This can be most realistically achieved with the physical presence of a human close to or within the trap. Therefore, replacing the human bait from the MET would reduce accuracy of the trap because other factors than the human odour, such as visual cues and body heat, are involved in attracting host-seeking mosquitoes [75, 76].

On a few occasions there was evidence of decreasing sensitivity of MET and CA-EG over the sampling night relative to the HLC, but this effect was not consistent between vector species, nor between indoor and outdoor settings. A reduction in the sampling efficiency of CA-EG relative to the HLC over the course of a night was reported in [49]. This was interpreted as a sign of battery drainage through time, which reduced the electrical output. Given that a decline in the relative sensitivity of electrocuting traps was not consistently reported in this study, it is difficult to interpret the patterns of time-dependent trap performance observed here. In addition to battery drainage, other factors, such as a build-up of moisture on traps (especially as occurred in outdoor stations) may have contributed to MET’s low relative performance. The MET output voltage was checked every hour and in some cases it was shown to drop below optimal levels, especially in the later hours of the night. Additionally, there were a few occasions where traps temporarily short-circuited during experiments because opposing wires came into contact, and/or the wooden frames became moist and mildly conductive. Experiments were stopped when there was an obvious cessation of current flow, however, there could have been more minor dips occurring during sampling night that went undetected. Use of a higher-capacity battery coupled to an alarm system to notify if and when there is any dip in electrical output could resolve any issues of variable voltage output through time.

This study shows no strong evidence of density-dependent sampling in either the MET or the CA-EG. However, this study was conducted over 21 consecutive nights in the rainy season when mosquito densities were generally high. Thus, it was not possible to assess density dependence across the full range of mosquito densities that occurs between wet and dry seasons. Additionally, it is noted that the detection of density dependence in trapping studies is sensitive to the type of analysis method used [77]. Several previous studies have assessed density dependency based on analysis of how the proportional catch rate varies with differing mosquito densities across nights [39, 78], whereas others, including the present study, use the Bland–Altman method [67]. The Bland–Altman method was chosen because its use of regression analysis to assess the reliability of two measures is not subject to bias inherent in the binomial, proportional catch approach. It is recommended that future studies to evaluate these trapping methods adopt a similar method so that estimations of density dependence are standardized and comparable.

For any mosquito-sampling tool to successfully replace the HLC, it must be able to give meaningful representation of key mosquito behaviours and associated human exposure risk factors. Here, three such measures were investigated that have been widely used in a number of other studies to assess both human risk and likely degree of protection from LLINs [15, 68–70, 79]. One of the most direct measures of indoor exposure is the proportion of mosquitoes that bite indoors (P_i_), for which comparable estimates were obtained from both electrocuting traps and the HLC. However, the proportion of mosquitoes caught when people are usually indoors (P_fl_) was underestimated by the CA-EG and MET for *An. gambiae s.l.*, but similar to the HLC for *An. funestus*. Estimates of the proportion of human exposure occurring indoors (π_i_) obtained from the CA-EG and MET were similar to the HLC for *An. gambiae s.l.*, but overestimated for *An. funestus.* This is consistent with results from a previous study [49] where the CA-EG produced a similar estimate of P_i_, but underestimated P_fl_ for *An. arabiensis* relative to the HLC. The likely explanation for this bias is the differential sampling efficiency of the electrocuting traps when used indoors versus out. This location-dependent performance would be expected to generate biased estimates of P_fl_ and the proportion of human exposure predicted to occur indoors.

Historical data for the Kilombero Valley (1999) where this study took place indicates the proportion of *An. gambiaes.l.* caught indoors (P_i_) estimated by HLC was 0.58 ± 0.01 [15], which is higher than the values of 0.37 ± 0.03 (HLC) and 0.35 ± 0.03 (MET) reported here. These differences may be due to concurrent changes within the *An. gambiaes.l.* complex that have occurred over this time. Whereas most *An. gambiae s.l.* were found to be *An. gambiae s.s.* in 1999 [54] to <1 % in 2009 [15], this species represents <1 % of the *An. gambiae s.l.* complex now with the remaining fraction being the more exophilic *An. arabiensis*. The proportion of human exposure occurring indoors that would otherwise be directly preventable with bed net use (π_i_) was estimated as 0.43 and 0.55 for *An. gambiaes.l.* and *An. funestuss.l.*, respectively, using HLC in this study. Assuming that all *An. gambiaes.l.* in this study were *An. arabiensis* (based on PCR results of 400 samples which showed all of them were *An. arabiensis*), these estimates of π_i_ are low compared to that reported in western Kenya [79], where values of 0.87 and 0.86 were obtained for *An. arabiensis* and *An. funestuss.l.*, respectively. A more recent study in western Kenya [80] reported π_i_ values of ~0.64 for major vectors *An. gambiaes.l.* and *An. funestus*, which are still higher but closer to the values reported in this setting. Another study in Dar es Salaam estimated π_i_ obtained for *An. arabiensis* to be 0.53 [49] which is also higher than found in this study. The consistently smaller values of π_i_ reported for both *An. gambiaes.l*. and *An. funestus s.l.* here indicates that a lower proportion of human exposure to malaria may be occurring indoors in the Kilombero Valley than in other parts of East Africa, and highlights the particular need for interventions that can control outdoor-biting mosquitoes in this setting.

As the MET applies high voltage electricity to electrocute mosquitoes, human safety in using this trap is a priority. Two measures were taken to ensure no risk of harm to humans using these traps. First, although the MET used relatively high pulsed DC voltage (600 V DC), resistors were incorporated to limit the current to no more than 10 mA which generates a low power output insufficient to cause harm to a human who momentarily touches them [81]. Similarly, although the CA-EG used higher voltages (800 V AC), resistors were used to limit current flow in this trap to 15 mA. A second measure can be incorporated into future versions of MET to remove even this mild risk of minor electrical sensation on contact by placing a protective barrier of non-conductive material in the inner side of the grids.

## Conclusions

This study has demonstrated proof-of-principle that the MET can be used with reasonable efficiency to sample malaria vectors outdoors. The CA-EG performance did not merit further consideration because of its low sampling sensitivity. Whereas the current version of MET may misrepresent some aspects of mosquito behaviour, such as the proportion of human exposure to biting that occurs indoors, it is hypothesized that the sampling sensitivity of MET can be improved specifically by ensuring generation of stable voltage across the night, and by avoiding short circuiting which can be achieved by replacing the semi-conducting wooden frames with non-conducting polyvinyl chloride (PVC). It is recommended further testing of the improved MET in a range of ecological settings to explore its ability to be used as an alternative to the HLC.

## Additional files

10.1186/s12936-015-1025-4 Details of Bland-Altman method for assessment of density dependency.
10.1186/s12936-015-1025-4 Correlation between the log-transformed [log(1+count)] number of mosquitoes caught by the HLC and those caught by the MET and the CA-EG. R is the Pearson’s correlation coefficient. P-values show the significance of the correlation between the number of mosquitoes sampled by HLC and that sampled by either MET or CA-EG.
