# Erratum to: Development and evaluation of mosquito-electrocuting traps as alternatives to the human landing catch technique for sampling host-seeking malaria vectors

**DOI:** 10.1186/s12936-016-1562-5

**Published:** 2016-11-15

**Authors:** Deodatus V. Maliti, Nicodem J. Govella, Gerry F. Killeen, Nosrat Mirzai, Paul C. D. Johnson, Katharina Kreppel, Heather M. Ferguson

**Affiliations:** 1 Institute of Biodiversity, Animal Health and Comparative Medicine, University of Glasgow, Graham Kerr Building, Glasgow, G12 8QQ UK; 2 Environmental Health and Ecological Sciences, Ifakara Health Institute, PO Box 78373, Kiko Avenue, Mikocheni B, Dar es Salaam, Tanzania; 3 Bioelectronics Unit, University of Glasgow, Graham Kerr Building, Glasgow, G12 8QQ UK; 4 Department of Vector Biology, Liverpool School of Tropical Medicine, Pembroke Place, Liverpool, L3 5QA UK; 5 School of Life Sciences, Nelson Mandela African Institute of Science and Technology Tanzania, PO Box 447, Arusha, Tanzania

## Erratum to: Malar J (2015) 14:502 DOI 10.1186/s12936-015-1025-4

The authors regret that the statistical analysis methods used in this article [1] to assess density-dependence between different types of trap (described in the third paragraph of the *Statistical analysis* section and in Supplementary Information S1) are invalid. However, re-analysis with a corrected method, described below, produced similar results to those obtained using the invalid method. Consequently, our conclusions regarding density-dependence are unchanged.

The methods in question aimed to test a null hypothesis of density-independence (linear correlation) against an alternative hypothesis of density-dependence (non-linear correlation), and to quantify the strength of linear correlation using the Pearson correlation coefficient. The flaw in the methods was in applying them to mosquito counts that had been log-transformed using ln(1 + count). This analysis assesses linearity on the log scale, but fails to take account of the fact that linearity on the log scale does not imply linearity on the untransformed count scale, which is the relevant scale when assessing density dependence. The simple solution of applying the same method to untransformed counts would not be valid due to violation of the standard assumptions of linear regression (normality and homoscedasticity of residuals), on which the method relies. We have therefore developed a new method for assessing density-dependence on the untransformed count scale, described fully in [2], which we have used to re-analyse the data in [1]. Briefly, the new method models density-dependence between two trapping methods as a power law relationship, with E(*x*_*i*_) = *α*E(*y*_*i*_)^*β*^, where *x*_*i*_ and *y*_*i*_ are the *i*th of *n* paired mosquito catches from traps of types X and Y, E(*x*_*i*_) and E(*y*_*i*_) are the expected counts of *x*_*i*_ and *y*_*i*_, and *α* is a scaling constant. The exponent *β* governs the degree of density-dependence [3, 4], so density-dependence can be quantified by the extent to which *β* deviates from 1. We calculated estimates and 95 % credible intervals (CI) for the density-dependence parameter, *β*, and a linear correlation coefficient for count data, *r*, defined in [2]. A 95 % CI for *β* that did not include 1 was taken as evidence for density-dependence. Both statistics were estimated using Markov chain Monte Carlo (MCMC) as described in [2], with the exception that, instead of 10^4^, a more informative prior variance for ln(*β*) of 1 was used (equivalent to specifying that, in the absence of data, the true value of *β* lies between 0.14 and 7.1 with 95 % probability). This change aided convergence when there was little information in the data about *β* due to low correlation between trapping methods.

Table 1 shows estimates and 95 % CIs quantifying the extent of density dependence (*β*) and linear correlation (*r*) between the mosquito electrocuting trap (MET) and human landing catch (HLC), and between the commercially available electrocuting grid (CA-EG) and HLC, for two species, *An. gambiae* s.l. and *An. funestus* s.l., collected indoors and outdoors (data from [1]). The *β* estimates and CIs replace the density-dependence results in Table 3 of [1], which were calculated using the invalid method. The new results are similar to the original results, in that none of the comparisons show evidence for density dependence, with all of the 95 % CIs including the null value of one. Similarly to the original results, the wide CIs for *β* support the interpretation that there was very low power to detect density-dependence. The linear correlation estimates (*r*) in Table 1 correspond closely to the estimates of Pearson’s correlation coefficient reported in Table S2 of [1] (mean absolute difference 0.05, maximum absolute difference 0.09), with the same three comparisons giving correlations significantly above zero. The five non-significantly correlated comparisons showed very wide CIs for *β*, of comparable width to the prior distribution, suggesting that, as expected when *r* is low, most or all of the information on *β* has come from its prior distribution.

**Table 1:** Results of the re-analysis of the density-dependence data with the corrected statistical analysis method, for comparison with Tables 3 and S2 in Maliti et al. [1]

Taxon	Location	Method	*β* estimate (95 % CI)	*r* estimate (95 % CI)
*An. gambiae* s.l.	Indoors	MET:HLC	1.17 (0.08, 2.83)	0.35 (0.00, 0.64)
		CA-EG:HLC	1.32 (0.05, 3.67)	0.22 (0.00, 0.54)
	Outdoors	MET:HLC	0.77 (0.32, 1.27)	0.59 (0.27, 0.86)^a^
		CA-EG:HLC	0.76 (0.22, 1.33)	0.51 (0.13, 0.83)^a^
*An. funestus* s.l.	Indoors	MET:HLC	1.35 (0.04, 4.02)	0.12 (0.00, 0.43)
		CA-EG:HLC	1.45 (0.03, 4.30)	0.13 (0.00, 0.47)
	Outdoors	MET:HLC	1.05 (0.62, 1.51)	0.76 (0.53, 0.94)^a^
		CA-EG:HLC	2.97 (0.06, 7.60)	0.15 (0.00, 0.54)

*MET* mosquito electrocuting trap, *HLC* human landing catch, *CA*-*EG* commercially available electrocuting grid

^a^Comparisons for which significant correlations were detected using the original methods

In summary, the conclusions regarding density-dependence that were reported in [1] were based on a flawed statistical analysis method, but are unchanged following re-analysis with a corrected method.
