# Supplementary material for: Development and evaluation of mosquito-electrocuting traps as alternatives to the human landing catch technique for sampling host-seeking malaria vectors
Source: Malar J. 2015 Dec 15;14:502. doi: 10.1186/s12936-015-1025-4 (PMC4681165; doi:10.1186/s12936-015-1025-4)
Supplement: Supplementary file 1 — 10.1186/s12936-015-1025-4 Details of Bland-Altman method for assessment of density dependency. [file 12936_2015_1025_MOESM1_ESM.docx]

**Supplementary information S1: Details of Bland-Altman method for assessment of density dependency**

We modeled density dependence as deviation from linear relationship between y - x and (x + y) / 2, where x and y are mosquito abundances from two different trapping methods recorded on the same night, transformed first by ln(abundance + 1) then standardized to have a mean = 0 and standard deviation = 1. Nonlinearity was modeled as a natural cubic spline with two degrees of freedom. A p-value for density dependence was estimated by comparing the null (linear) model with the density dependent model (nonlinear) using a likelihood ratio test. Density dependence was quantified by calculating the adjusted *R*^2^ (*R*^2^_adj_) of the nonlinear model relative to the linear model as *R*^2^ - (1 - *R*^2^) p / (n - p - 1), where p = 2, the number of degrees of freedom of the spline. In these analyses, a high value of *R*^2^_adj_ indicates density dependence, while a low value can arise from either density independence, or high variability in the data set with limits statistical power.
