# Supplementary material for: Development and evaluation of mosquito-electrocuting traps as alternatives to the human landing catch technique for sampling host-seeking malaria vectors
Source: Malar J. 2015 Dec 15;14:502. doi: 10.1186/s12936-015-1025-4 (PMC4681165; doi:10.1186/s12936-015-1025-4)
Supplement: Supplementary file 2 — 10.1186/s12936-015-1025-4 Correlation between the log-transformed [log(1+count)] number of mosquitoes caught by the HLC and those caught by the MET and the CA-EG. R is the Pearson’s correlation coefficient. P-values show the significance of the correlation between the number of mosquitoes sampled by HLC and that sampled by either MET or CA-EG. [file 12936_2015_1025_MOESM2_ESM.docx]

**Table S2:** Correlation between the log-transformed [log(1+count)] number of mosquitoes caught by the HLC and those caught by the MET and the CA-EG. R is the Pearson’s correlation coefficient. P-values show the significance of the correlation between the number of mosquitoes sampled by HLC and that sampled by either MET or CA-EG.

| Taxon |  | Location |  | Method |  | R |  | P-value |
| --- | --- | --- | --- | --- | --- | --- | --- | --- |
| *An. gambiae* s.l. |  | Indoors |  | MET:HLC |  | 0.35 |  | 0.12 |
|  |  |  |  | CA-EG:HLC |  | 0.28 |  | 0.21 |
|  |  | Outdoors |  | MET:HLC |  | 0.65 |  | 0.001 |
|  |  |  |  | CA-EG:HLC |  | 0.58 |  | 0.005 |
| *An. funestus* s.l. |  | Indoors |  | MET:HLC |  | 0.18 |  | 0.43 |
|  |  |  |  | CA-EG:HLC |  | 0.12 |  | 0.59 |
|  |  | Outdoors |  | MET:HLC |  | 0.67 |  | <0.001 |
|  |  |  |  | CA-EG:HLC |  | 0.22 |  | 0.32 |
